# Maternal depression trajectories and child BMI in a multi-ethnic sample: a latent growth modeling analysis

**DOI:** 10.1186/s12884-021-04308-0

**Published:** 2021-12-13

**Authors:** Charlotte V. Farewell, Ryley Donohoe, Zaneta Thayer, James Paulson, Jacinda Nicklas, Caroline Walker, Karen Waldie, Jenn A. Leiferman

**Affiliations:** 1 Department of Community and Behavioral Health, Colorado School of Public Health, University of Colorado – Anschutz Medical Campus, 13001 East 17th place Mail Stop B119, Aurora, CO 80045 USA; 2 Dartmouth College, Hanover, NH USA; 3 Old Dominion University, Norfolk University, Norfolk, VA USA; 4 University of Auckland, Auckland, New Zealand

**Keywords:** Perinatal mental health, Perinatal depression, Latent growth modeling, Child body mass index, Antenatal depression

## Abstract

**Background:**

Perinatal (antenatal and postpartum) depression impacts approximately 12% of mothers. Perinatal depression can impact everyday functioning for mothers, and the relationship with, and development of, their children. The purpose of this study was to investigate depression trajectories from the antenatal period through 54-months postpartum and associations with child body mass index at 54-months postpartum.

**Methods:**

This study applied latent growth modeling to the Growing Up in New Zealand study, which is a longitudinal pregnancy cohort study that provides nationally representative-level data, to investigate associations between depression at three time points (antenatal, 9-months postpartum, 54-months postpartum) and child body mass index at 54-months (*n*=4897).

**Results:**

The average slope of depression for this sample is low and decreases over time. When child BMI was added to the model as an outcome variable, both antenatal depression (B=.25, *p*<.01), and the rate of change of depression across the perinatal and postpartum periods (B=.09, *p*<.01) were associated with child BMI at 54-months postpartum. After controlling for sociodemographic characteristics, antenatal depression, but not the slope of depression, remained significantly associated with child BMI (B=.05, *p*<.05). When controlling for maternal pre-pregnancy BMI the effect of antenatal depression on child BMI at 54-months was entirely attenuated (*χ*^2^ (9) = 39.60, *p* < .05, SRMR = 0.01, CFI = .99, RMSEA = 0.03, BIC=53213).

**Conclusions:**

Our findings align with the Developmental Origins of Health and Disease theory and imply that both the physical and mental health of mothers during pregnancy may be important indicators of child growth and development outcomes. Early intervention directed towards women who have even mild depression scores during pregnancy may promote healthy child development outcomes. Additionally, given the heterogeneity of depressive symptoms over time seen in this study, multiple assessment periods across the postpartum period may be valuable to adequately address and support maternal mental health.

## Introduction

Perinatal depression (antenatal and postpartum) is defined symptomatically as exceeding a threshold on a screening measure, such as the Edinburgh Postnatal Depression Scale [1]. Studies vary in prevalence based on the timing and method of perinatal depression measurement, but estimates suggest that perinatal depression impacts approximately 12% of mothers [2]. In a recent systematic review, rates of depression appeared to be higher during pregnancy (17%) compared to the postpartum period (13% )[3]. Sixty-one percent (61%) of mothers with antenatal depression do not experience postpartum depression, and 53% of mothers with postpartum depression did not have depression during pregnancy [3]. Mothers who suffer from persistent depression (antenatal and postpartum) are at particular risk for negative maternal and child health outcomes [4].

Perinatal depression can impact everyday functioning for mothers, and the relationship with, and development of, their children [5]. For example, perinatal depression has been associated with an increased risk for early childhood depression, impaired or delayed language, cognitive development and behavioral problems [6]. Chronic perinatal depression has also been linked to poor social emotional development in early childhood [7]. One study found that children whose mothers experienced perinatal depression had lower social skills at 5 years of age [8]. Poor development and depression early in life can lead to disparities in physical, emotional and mental health outcomes throughout the life course [9]. Given the importance of childhood wellbeing throughout the life course, it is imperative to investigate the relationship between perinatal depression and its impact on early childhood development outcomes.

Maternal depression during the perinatal periods has also been positively associated with obesity in early childhood; however, the findings vary by timing and duration of exposure [10–12]. Elevated body mass index (BMI) is a particularly important outcome of interest, since high BMI in childhood is a major risk factor underlying the development of many chronic diseases [13]. Identifying the determinants of obesity within a broader multi-level framework emphasizes the upstream influences of maternal mental health and modifiable factors beginning very early in the life course [14]. Promoting maternal mental health during the perinatal period, and beyond, may foster women’s wellbeing and promote healthy childhood growth and development outcomes [15, 16].

Two different theoretical models describe the potential mechanistic pathways (or developmental trajectories) of maternal depression on childhood development outcomes: the Developmental Origins of Health and Disease (DOHaD) model [17, 18] and the Life Course Health Development (LCHD) model [19]. The DOHaD model explores the biological embedding of adversity (e.g., maternal depression) primarily during the antenatal period of development [20]. The LCHD framework incorporates multiple theories, including DOHaD, and contends that health is a consequence of cumulative factors that change as a person develops throughout the life course [21]. Studies have found that both antenatal and cumulative perinatal depression are associated with poor child outcomes [11]. However, the research is often cross-sectional, retrospective, relies on subjective or un-validated measures, and/or is limited to the first two years of life [10, 11].

Cross-sectional methods to investigate perinatal depression do not adequately address the timing and duration of maternal mood and resulting impacts on maternal and child wellbeing [3]. In contrast, the use of longitudinal data, and latent growth modeling in particular, allows for the investigation of both the intercept and slope of maternal depression from the antenatal period through five-years postpartum and associations with early childhood outcomes. These methods can be used to explicate the ways in which adversities, such as depression, may aggregate over time and affect the intergenerational transmission of poor mental and physical health outcomes.

Additionally, a recent systematic review of perinatal depressive symptom trajectories emphasized the need for further research within larger and more diverse settings [22]. Specifically, there is a lack of evidence on maternal depression among minority groups within broader populations and those from lower socioeconomic backgrounds [23]. To address this need, this study applied latent growth modeling to the Growing Up in New Zealand (GUiNZ) study, which is a longitudinal pregnancy cohort study that provides nationally representative-level data. The GUiNZ study is unique in terms of the size and capacity to provide a comprehensive picture of maternal mental health and child development across multiple domains of influence, and for the inclusion of significant numbers of minorities, representative of this multicultural population. The purpose of this study was to investigate depression trajectories from the antenatal period through 54-months postpartum and their associations with child BMI at 54-months postpartum.

## Methods

### Participants

Participants for this study came from the GUiNZ study [24]. The GUiNZ study included pregnant persons with an expected delivery date between April 2009 and March 2010 residing within a geographically defined region of New Zealand, chosen for its nationally representative ethnic and socioeconomic diversity. The cohort originally consisted of 6,853 children live-born to 6,822 women (11% of the national birth cohort over the recruitment period) and has been demonstrated to be representative of the broader current New Zealand birth population [24]. To date, there have been five waves of data collection (e.g., antenatal, 9-months, 24-months, 54-months, 8-years). Ethical approval for this study was obtained from the Colorado Institutional Review Board and the Ministry of Health Northern Y Regional Ethics Committee (NTY/08/06/055) in New Zealand. Three time points of data were used for the current study: T1 (pregnancy), T2 (9-months postpartum), and T3 (54-months postpartum). Depression data was not collected during the 24-month data collection wave.

### Instruments

#### Outcome Variable: Child BMI z-score at 54-months

Child BMI was calculated as weight in kilograms divided by height in meters squared measured at the 54-months interview; children ranged in age from 48- to 60-months. The BMI scores were z-scored based on the continuous measure of gender- and age-specific BMI z-scores from the International Obesity Task Force (IOTF) Growth Standards [25].

#### Independent Variable: Antenatal and Postpartum Depression

Depression symptoms were measured during the antenatal and 9-month interviews using the Edinburgh Postnatal Depression Scale (EPDS) [1], a screening tool which consists of 10 self-report items focused on the cognitive and affective features of depression. Although the EPDS was originally designed to screen for postnatal depression, it has been validated in populations of pregnant women [26]. Depression at 54-months postpartum was assessed using the Patient Health Questionnaire 9-item (PHQ-9) [27]. All depression scores were standardized using z-scores at each timepoint to allow for comparison between measures.

#### Covariates

Socio-demographic variables were collected by self-report during the antenatal interview and included maternal age (years), education level (no secondary school qualification, secondary school degree, diploma or trade certificate, bachelor's degree, higher degree), total household income (<$20,000, $20,001-$30,000, $30,001-$50,000, $50,001-$70,000, $70,001-$100,000, $100,001-$150,000, >$150,001) and ethnicity (see below). Maternal age, education level, and total household income were modeled continuously. Ethnicity was defined as the woman’s self-prioritized ethnicity (New Zealand European; Māori; Pacific Peoples; Asian; Middle Eastern, Latin American and African (MELAA); and other (Statistics New Zealand 2004). External prioritization was carried out (as utilized by Statistics New Zealand 2004) for mothers who identified with more than one ethnicity but who did not provide a self-prioritized main ethnicit y[28]. In this study participants identifying as MELAA or other were grouped together as Other ethnicities (*n*=141). Maternal ethnicity was coded as a categorical variable in all analyses and New Zealand European was used as the reference category (1=New Zealand European, 2= Māori, 3=Pacifika, 4=Asian, 5=Other). Maternal pre-pregnancy BMI was collected via self-reported height and weight during the antenatal interview and was included in all analyses as a continuous variable.

### Analysis

GUiNZ data sets from three data collection waves, antenatal, 9-months and 54-months, were merged using the unique child identifier. Individuals who had non-missing data with respect to childhood BMI at 54 months variable were included (*n*=5839). Within this sample, only mothers who completed an antenatal interview during pregnancy (depression data collected during pregnancy) were included for the final analytical sample (*n*=4897). More than 5% of data were missing on key variables (range from 0% to 11.3%) so multiple imputation was used to create imputed values for missing data across all variables. Although little MCAR’s test was significant (*χ*^2^ (128) = .686.58, *p*<.05), a missing at random assumption was used and the imputation model included multiple sociodemographic variables that may be predictive of missing data in each covariate, as well as the outcome variable, in the final latent growth model. Additionally, the proportion of missing data were not deemed to be too large, the proportion of missing data for the outcome variable was very low (1.7%), and the observed cases and the imputed cases were analyzed separately to compare results. Descriptive statistics and findings were not different suggesting that these data were missing at random and it was therefore appropriate to use multiple imputation methods [29, 30].

First, we ran univariate and bivariate statistics to explore all key variables. Next, we used latent growth curve modeling to investigate relationships between depression at three timepoints and child BMI, controlling for covariates. The following four models were run: 1) Unconditional two-factor (intercept, slope) linear growth model with varying fixed factor loadings; 2) exploratory models assessing for nonlinear quadratic trends in the trajectory; 3) analysis of outcomes (i.e., child BMI) of the intercept and slope; and 4) analysis of potential covariates (i.e., age, education, income, ethnicity, maternal pre-pregnancy BMI). Models were estimated and compared using well-established criteria for model fit to determine the best-fitting model including the chi square, Standardized Root Mean Square Residual (SRMR) (<.05), Comparative fit index (CFI) (>.95), Root Mean Square Approximation (RMSEA) (<.06), and the Bayesian Information Criteria (BIC) (lower value indicates better fit model). Models were inspected for convergence, replication of the best log likelihood, and entropy. Standardized coefficients, standard errors, and fit statistics are presented for all final models. All analyses were conducted with Mplus Version 8 [31] and analyses were considered significant at p<.05.

## Results

Characteristics of the analytical sample are displayed in Table 1. The average child BMI

**Table 1:** Descriptive Characteristics of Analytical Sample (*n*=4897)

Variables	Analytical Sample (*n*=4897)		
		m	sd
Child Body Mass Index at 54-months		0.76	1.1
Antenatal Depression (EPDS)		6.0	4.9
9-months Depression (EPDS)		5.1	4.5
54-Months Depression (PHQ-9)		3.5	3.7
Maternal Age		30.4	5.7
Maternal Pre-Pregnancy BMI		25.3	5.7
		**n**	**%**
Maternal Self-Identified Ethnicity			
	European	2949	60.2
	Māori	587	12.0
	Pacifika	509	10.4
	Asian	589	12.0
	Other	263	5.4
Education			
	No sec school qualification	267	5.5
	Sec school/NCEA 1-4	1058	21.9
	Diploma/Trade cert/NCEA 5-6	1469	30.4
	Bachelor’s degree	1180	24.5
	Higher degree	852	17.7
Household Income			
	<$20,001	145	3.5
	$21,001-$30,000	196	4.7
	$30,001-$50,000	524	12.5
	$50,001-$70,000	663	15.9
	$70,001-$100,000	978	23.4
	$100,001-$150,000	1010	24.2
	>$150,001	660	15.8

IOTF z-score at 54 months was 0.76. The mean antenatal and 9-months postpartum depression score based on the EPDS were 6.0 and 5.1, respectively; the average 54-months postpartum depression score based on the PHQ-9 was 3.5. On average, mothers were 30 years of age with a BMI of 25. The ethnicity of the sample participants consisted of 60% New Zealand European, 12% Maori, 10% Pacifika, 12% Asian, and 5% as other. Almost half of the sample had at least a bachelor’s degree (42%) and 40% reported an annual household income of at least $100,000 NZD.

All variables included in the final model were significantly correlated (see Table 2). Antenatal depression scores were moderately correlated with postpartum depression scores at 9-months (r=.45, *p*<.01) and 54-months (r = .30, *p*<.01). Depression scores at 9-months and 54-months were also moderately correlated (r=.33, *p*<.01). Antenatal, 9-months and 54-months postpartum depression scores were inversely correlated with education level (r ranges from -.12 to -.15, *p*<.05), household income (r ranges from -.17 to -.19, *p*<.05), and maternal age (r ranges from -.13 to -.19, *p*<.05). There was a small, but significantly positive correlation between maternal pre-pregnancy BMI and depression scores at each of these three time points (r=.10, .07, .13; all *p*<.01). Child BMI at 54-months was significantly correlated with depression at all three time points (range of r=.06-.08, *p*<.01), household income (r=-.13, *p*<.01), educational attainment (r=-.17, *p*<.01), age (-.09, *p*<.01), and pre-pregnancy BMI (r=.31, *p*<.01).

**Table 2:** Correlations between continuous variables (*n*=4897)

Variables		1	2	3	4	5	6	7	8
1	Antenatal Depression	-							
**2**	9-months Depression	0.45**	-						
**3**	54-months Depression	0.30**	0.33**	-					
**4**	Child BMI at 54-mos	0.07**	0.06**	0.08**	-				
**5**	Household Income	-0.19**	-0.18**	-0.17**	-0.13**	-			
**6**	Educational Attainment	-0.15**	-0.12**	-0.14**	-0.17**	0.40**	-		
**7**	Maternal Age	-0.19**	-0.13**	-0.16**	-0.09**	0.38**	0.33**	-	
**8**	Pre-pregnancy BMI	0.10**	0.07**	0.13**	0.31**	-0.13**	-0.21**	-0.04	-

*p<.05

**p<.01

Table 3 presents results of growth models predicting the effect of baseline and trajectory of depression on child BMI at 54-months postpartum. The linear growth model with fixed factor loadings for the three timepoints at 0, 1, and 3, respectively, was deemed to be the best fit model compared to the no change models and nonlinear models based on model fit criteria (*χ*^2^ (1) = .021, *p* > .05, SRMR = 0.001, CFI = 1.00, RMSEA = 0.00, BIC=39546). Consistent with life course research on depression trajectories, results from the linear growth models indicate that the average slope of depression for this sample is low and decreases across time. When child BMI was added to the model as an outcome variable, both antenatal depression (B=.25, *p*<.01), and the rate of change of depression across the perinatal and postpartum periods (B=.09, *p*<.01) were associated with child BMI at 54-months postpartum (Model 1).

**Table 3:** Latent Growth Model Predicting Child BMI by Antenatal Depression (intercept) and the Trajectory of Depression (slope) among mothers from the Growing Up in New Zealand Data Set (*n*=4879)

Variables		Model 1		Model 2		Model 3	
		Beta	SE	Beta	SE	Beta	SE
Antenatal Depression (Intercept)		0.15**	0.02	0.05*	.02	0.02	0.42
	Income			-0.14**	.02	-0.14**	.02
	Education			-0.05**	.02	-0.05**	.02
	Age			-0.14**	.02	-0.14**	.02
	Maori			0.07**	.02	0.07**	.02
	Pacifika			0.15**	.02	0.14**	.02
	Asian			0.03	0.02	0.04	0.02
	Other			0.02	0.40	0.02	0.40
	Pre-Pregnancy BMI					0.04*	.02
Depression Trajectory (Slope)		0.09**	0.04	0.05	0.17	0.02	0.62
	Income			-.00	0.88	-.00	0.03
	Education			-0.01	0.03	-0.01	0.03
	Age			0.03	0.36	0.02	0.36
	Maori			-0.01	0.79	-0.02	0.79
	Pacifika			-0.12**	.00	-0.13**	.00
	Asian			-0.10**	.03	-0.08*	.03
	Other			-0.05	0.06	-0.05	0.06
	Pre-Pregnancy BMI					0.08*	.03
BIC		54429.63		53559.86		53330.81	
CFI		1.00		0.99		0.99	
SRMR		.00		0.01		0.01	
RMSEA		.00		.03		.03	

After controlling for sociodemographic characteristics (Model 2), antenatal depression, but not the slope of depression, remained significantly associated with child BMI (B=.05, p<.05), though the relationship was attenuated. Having lower income, education, and age were associated with higher antenatal depression scores (B=-.14, -.05, -.14, respectively; all *p*<.01). Additionally, lower income and education were associated with higher levels of child BMI at 54-months postpartum (B=-.05, -.06, respectively; both *p*<.01). When controlling for maternal pre-pregnancy BMI (Model 3), the effect of antenatal depression on child BMI at 54-months was entirely attenuated (*χ*^2^ (9) = 39.60, *p* < .05, SRMR = 0.01, CFI = .99, RMSEA = 0.03, BIC=53213). Maternal BMI was significantly and positively associated with antenatal depression scores (B=.04, *p*<.05), the slope of perinatal depression (B=.08, *p*<.05), and child BMI at 54-months postpartum (B=.21, *p*<.01).

**Fig. 1:**
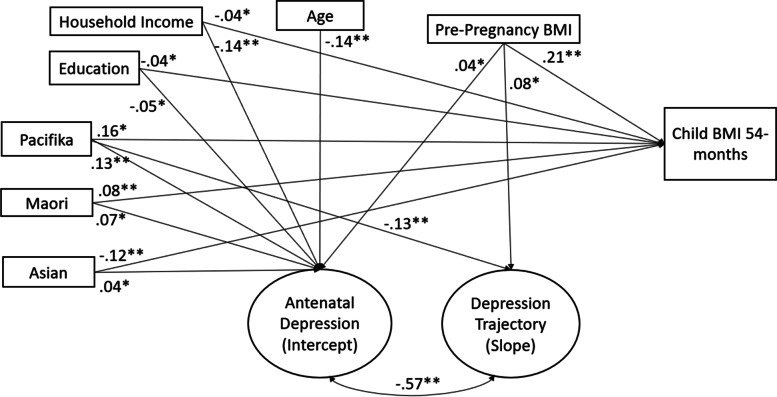
A latent growth model highlighting significant associations between antenatal depression (intercept), the trajectory of depression from the antenatal period through 54-months postpartum (slope), and child BMI at 54-months, controlling for sociodemographic factors and maternal pre-pregnancy BMI. *p<.05. **p<.01

Results from the full model are presented in Fig. 1.

## Discussion

The purpose of this study was to apply a multivariate latent growth curve model to a large, longitudinal and representative data set in New Zealand to examine the relationships between depression during early critical periods (e.g., prenatal, postpartum and parenting in early childhood) and child BMI at 54-months postpartum. Though antenatal, 9-months, and 54-months depression scores were moderately correlated (r ranged from .30-.45), the fit of the linear change model of depression was better than that of no change model, suggesting that on average, maternal depression scores are heterogeneous over time in this sample [32–34]. Our findings also suggest that depression scores generally decrease from the antenatal period through 54-months postpartum. These findings are supported by recent studies that have investigated changes in depression across these transitional periods [35, 36].

Prior to controlling for covariates, both the intercept *and* slope of maternal depression was associated with child BMI at 54-months postpartum. These data support the LCHD framework [19], suggesting that the cumulative effect of poor maternal health has detrimental impacts on child growth above and beyond exposure solely during the antenatal period. However, after controlling for sociodemographic factors, the association between the slope of depression and child BMI at 54-months was attenuated. In line with the DOHaD framework and past literature, a mother’s depressive symptomology during pregnancy may detrimentally impact early childhood BMI, regardless of postpartum maternal mental health status [37–39]. Education, income and age were inversely associated with both antenatal depression and early childhood BMI; age and household income were the strongest predictors of antenatal depression scores in this sample. These findings support previous literature on the impact of sociodemographic factors on antenatal depression scores and subsequent increases in the risk of adverse childhood outcomes [40]. Studies have found that education level and income are negatively associated with perinatal depression [41] and mothers having children at a younger age are at a higher risk for antenatal depression [42]. Sociodemographic factors were also significantly associated with child BMI; lower socioeconomic status was significantly associated with higher child BMI, potentially due to the structural factors (e.g., food access, affordability) that impact child growth and development outcomes [43]. Variations in depression and child BMI by ethnic group were identified as statistically significant. Pacifika, Maori, and Asian mothers reported significantly higher depression scores during the antenatal interview compared to New Zealand European mothers, with Pacifika individuals reporting the highest scores, which aligns with past studies with the GUiNZ data set [3]. Children of Pacifika and Maori mothers had higher BMIs compared to New Zealand European children [44].

After controlling for all sociodemographic factors, only antenatal depression scores were associated with child BMI; depression throughout the postpartum period was no longer statistically significantly related to the outcome. A systematic review cited some studies that contradicted our findings and found a significant positive association between chronic perinatal and postpartum depression and risk factors for children being overweight in preschool, after controlling for covariates of socioeconomic status, maternal education status, marital status and ethnicity of the child [45]. However, the results were inconsistent across studies and depended on the time at which depression was measured (i.e., antenatal, postnatal, in isolation or longitudinally). In the GUiNZ sample, depression scores during the antenatal period appear to be most strongly associated with child BMI, while depression in the postpartum period did not contribute additional risk [39].

When maternal BMI was added into the final model, the association between depression and child BMI at 54-months of age was no longer statistically significant. Though this is supported by past studies [46], substantial heterogeneity in findings with respect to perinatal depression and child BMI z-scores exist [10]. For example, one recent study found that perinatal depression was associated with higher BMI for the child at three years of age, even after controlling for maternal BMI and childbirth weight [39]. These discrepancies may be partially explained by the interaction of mental and physical health during the antenatal period [47], which may amplify negative impacts on child growth and development outcomes. In the current sample, there was a small, though significant, correlation between antenatal depression and pre-pregnancy BMI. An earlier study found that mothers who were depressed *and* had a high BMI had infants with a higher birth weight compared to mothers who were not depressed and did not have a high BMI [48]. Collectively, these findings confirm that strong associations exist between physical and mental health outcomes and that physical health, and specifically maternal weight, may be the biggest predictor of early childhood BMI above and beyond the effects of depression on this outcome [49].

Despite the large sample size and longitudinal nature of the study, this study is not without limitations. Exposure to antenatal and postpartum depression is not necessarily causally related to early childhood BMI. In addition, many of the effect sizes were weak, but significant due to the large sample size. As such, these significant findings may not be clinically significant. Future research is needed to continue exploring the biological and behavioral pathways that may create these associations. Both perinatal depression and early childhood obesity are complex health conditions. A variety of different risk and resilience factors may influence the observed associations. Depression instruments were not consistent across timepoints; since the EPDS has an anxiety subscale and the PHQ-9 does not, higher scores during the first two timepoints may reflect anxiety, in addition to depression, which may have accounted for decreasing depression scores over time. Additionally, our slope variances were small and simulation studies have suggested fit problems with low slope variances; however, fit for the current models was achieved with no error (no convergence problems) [50]. Finally, maternal BMI was self-reported, which may represent a biased estimate, and we assumed that baseline covariates in our analyses (e.g., income, education) were stable over time.

Previous research examined trajectories between antenatal depression and depression up to 1 to 2 years postpartum, but very few studies investigate trajectories in maternal depression through early childhood [36, 51]. Strengths of this research include the use of validated screening tools, the large sample size and ethnic and socioeconomic diversity of the GUiNZ sample, the low attrition rates, and the investigation of data from the antenatal period through 54-months postpartum. Although our findings align with the DOHaD theory and imply that both the physical and mental health of mothers during pregnancy may be important indicators of child growth and development outcomes, antenatal depression and the slope of depression through 54-months postpartum were moderately and significantly correlated and depression scores changed over time Mental health supports and resources that begin during pregnancy can impact depression trajectories throughout the life course, and therefore may be particularly protective. In support of the LCHD framework, multiple points of intervention may be imperative to maintain positive mental health throughout the postpartum and early childhood periods. To further investigate and compare these frameworks, additional child outcomes that may be more closely related to maternal depression should be explored.

Early intervention directed towards women who have even mild depression scores during pregnancy may promote healthy child development outcomes. However, these findings suggest that both sociodemographic factors and maternal pre-pregnancy BMI may be stronger predictors of child BMI compared to maternal depression during the prenatal and/or postpartum periods. Early childhood obesity interventions that are targeted towards these factors, such as exercise promotion programs, may yield greater benefits compared to mental health-focused interventions during the prenatal or postpartum periods. Additionally, given the heterogeneity of depressive symptoms over time seen in this study, multiple assessment periods across the postpartum period may be valuable to adequately address and support maternal mental health. Finally, there is a need to apply innovative modeling techniques, such as growth mixture modeling, to these types of data to investigate cohorts of women who may follow similar trajectories with respect to maternal mental health and allocate resources towards highest risk individuals. Differentiating public health and clinical health supports during these early sensitive periods may mitigate disparities between maternal and child mental and physical health outcomes.

## Data Availability

The datasets used and/or analysed during the current study are available from the corresponding author on reasonable request.

## Abbreviations

GUiNZ: Growing Up in New Zealand
BMI: Body Mass Index
IOTF: International Obesity Task Force
DOHaD: Developmental Origins of Health and Disease
LCHD: Life Course Health Development
EPDS: Edinburgh Postnatal Depression Scale
PHQ-9: Patient Health Questionnaire
SRMR: Standardized Root Mean Square Residual
CFI: Comparative fit index
RMSEA: Root Mean Square Approximation
BIC: Bayesian Information Criteria
